# Psychological Quarterly Retrospect

**Published:** 1860-07-01

**Authors:** 


### *Psychological Quarterly Retrospect.*

We should either have been more or less than man if we had escaped the infectious enthusiasm of that glorious movement which may be said to have attained maturity in the Volunteer Review, before Her Majesty, on the 23rd ult. We refer to the movement here, not that we propose to discuss its ethical or æsthetical aspects. These are sufficiently impressed upon the feelings of every true Englishman. But there is an incidental circumstance connected with the growth of the Volunteer Rifle Corps, which is worthy of being called to mind, as being amusingly illustrative of the little change which man's habits of thought undergo in the course of ages. "My good sir," exclaims *Paterfamilias*, seizing us warmly by the arm, if we happen to stray into the drill-ground, or to be his neighbour at the dinner-table, or to come across him in the Park—"My good sir," he exclaims, "now isn't this truly excellent for our young men; this drill, this rifle exercise, this so forth." Of course Paterfamilias never touches upon the great moral and political significations of the Rifle movement. He knows that to be useless. He feels that he and I are of the fullest accord in all that relates to its greatness, its goodness, and its significant meaning across the water. He knows very well that if he and I could get over the impertinent simper of our tailor if we alluded to the matter, and the tale-telling of our bare-faced cheval-glass, we should both endue our very civilian-like bodies in the grey or the green of a Volunteer Corps before sunset, and amble into the goose-step with the most serious gravity. He knows all this, and consequently he keeps to other and by no means uninteresting matter. "What can be better," he proceeds, "than this constant drill, this wielding a ponderous rifle (that is, as you of course understand, comparatively speaking), for strengthening the frame, developing the brawn, my dear sir; giving a firmer, a nobler, an altogether better gait, sir; weaning the unsophisticated youth from more dangerous pleasures—yes, sir, pleasures: I mean what I say, and I assert that every volunteer ranks his duty among his greatest pleasures. Now, sir, you are a medical man, and I should like to hear what you think of this view of the question." Thus Paterfamilias; and we assent most warmly to his opinions, and confirm them by showing to him that they possess all the respectability, all the stability which may be derived from hoar antiquity.

Turn for a moment to Plato's dialogue concerning Courage—the

Laches. Lysimachus and Melesias, two of the interlocutors, are country gentlemen. They are anxious about the education of their sons, and have been recommended to let them learn a certain sword-exercise. They consult Nicias and Laches, two eminent military men at Athens, and it is in this fashion (to quote Dr. Whewell's summary[1]) that Nicias gives his opinion in favour of the sword-exercise :—

"It keeps young men out of worse employments of their leisure, gives them strength and agility, is a preparation for actual war, both in the rank and single affrays ; and is likely to set young men upon learning other parts of the art of war." It would also, he says, make a man braver and bolder than he would otherwise be ; and a thing, he says, not to be despised, would give him a military carriage which would inspire awe. "So that," he says, in conclusion, "I think, and for these reasons, that it is a good thing to teach the young men this exercise."

And so it is, also, we echo, and with truth, in no small degree, of the drill and discipline of our Volunteer Corps, and we doubt not that these will, henceforth, play a not unimportant part in the moral and physical education of our youth ; and this without putting to flight the more sober civilian virtues upon which we are apt to pride ourselves as a nation.

And now, if we would learn another lesson from the passing events of the quarter, let us turn to Mr. Gladstone's address upon his installation as Rector of the University of Edinburgh. Comment would be almost impertinent upon the noble language and nobler thoughts of the trumpet-tongued speaker :—

"You have chosen, gentlemen, as your own representative in the University Court one widely enough separated from you in the scale of years ; one to whom much of that is past which to you is as yet future. It is fitting, then, that he should speak to you on such an occasion of that which unites us together—namely, the work of the University, as a great organ of preparation for after life ; and that, in treating of what constitutes the great bond between us, he should desire and endeavour to assist in arming you, as far as he may, for the efforts and trials of your career. Subject to certain cycles of partial revolution, it is true that, as in the material so in the moral world, every generation of man is a labourer for that which succeeds it, and makes an addition to that great sum total of achieved results which may, in commercial phrase, be called the capital of the race. Of all the conditions of existence in which man differs from the brutes there is not one of greater moment than this, that each one of them commences life as if he were the first of a species, whereas man inherits largely from those who have gone before. How largely, none of us can say ; but my belief is that, as years gather more and more upon you, you will estimate more and more highly your debt to preceding ages. If, on the one hand, that debt is capable of being exaggerated or misapprehended, if arguments are sometimes strangely used which would imply that, because they have done much, we ought to do nothing more ; yet, on the other hand,

[1] *The Platonic Dialogues*, Vol. I., p. 20.

it is no less true that the obligation is one so vast and manifold that it can never as a whole be adequately measured. It is not only in possession available for use, enjoyment, and security; it is not only in language, laws, institutions, arts, religion; it is not only in what we have, but in what we are. For, as character is formed by the action and reaction of the human being and the circumstances in which he lives, it follows that as those circumstances vary he alters too, and he transmits a modified—it ought to be also an enlarged and expanding—nature onwards in his turn to his posterity, under that mysterious law which establishes between every generation and its predecessors a moral as well as a physical association. In what degree this process is marred, on the one hand, by the perversity and by the infirmity of man, or restored and extended, on the other, by the remedial provisions of the Divine mercy, this is not the place to inquire. The progress of mankind is, upon the whole, a chequered and an intercepted progress; and even where it is full-formed, still, just as in the individual, youth has charms that maturity under an inexorable law must lose, so the earlier ages of the world will ever continue to delight and instruct us by beauties that are exclusively or peculiarly their own. Again, it would seem as though this progress (and here is a chastening and a humbling thought) were a progress of mankind and not of the individual man, for it seems to be quite clear that, whatever be the comparative greatness of the race now and in its infant or early stages, what may be called the normal specimens, so far as they have been made known to us either through external form or through the works of the intellect, have tended rather to dwindle, or at least to diminish, than to grow in the highest elements of greatness. But the exceptions at which these remarks have glanced neither destroy nor materially weaken the profound moment of the broad and universal canon, that every generation of men, as they traverse the vale of life, are bound to accumulate, and in divers manners do accumulate, new treasures for the race, and leave the world richer, on their departure, for the advantage of their descendants, than, on their entrance, they themselves have found it. Of the mental portion of this treasure no small part is stored; and of the continuous work I have described no small part is performed by Universities, which have been, I venture to say, entitled to rank among the greater lights and glories of Christendom." (Applause.)

Mr. Gladstone then proceeded to describe the idea and work of an University somewhat in detail, and he concluded his address in these words:—

"And now, my younger friends, you to whom I owe the distinction of the office which enables and requires me to address you, if I have dwelt thus at length upon the character and scope of Universities and their place in the scheme of Christian civilization, it is in order that, setting before you the dignity that belongs to them, and that is reflected on their members, and the great opportunities which they offer both of advancement and of improvement, I might chiefly suggest and impress by facts, which may be more eloquent than precepts, the responsibilities that are laid upon you by the enjoyment of these gifts and blessings. Much, however, might be said to you on the acquisition of the knowledge which will be directly serviceable to you in your several professions; much on the immense value of that kind of training in which the subjects learned have for their chief aim not to inure the hand (so to speak) to the use of its tools in some particular art, but to operate on the mind itself, and, by making it flexible, manifold, and strong, to endow it with a general aptitude for the duties and exigencies of life; much, lastly, on the frame of mind in which you should pursue your work. Of these three branches, the topics belonging to the first are the most obvious and simple, for it requires no argument to persuade the workman that he must be duly furnished with his tools and must know how to handle them. The means are less directly palpable which have made it the habit of our country to spend, where means permit, many precious years upon studies void in a great degree of immediate bearing upon the intended occupations of our after life. Those may, however, be the means of showing, first, that even the direct uses of the studies which you include under the general designation of humanity are more considerable when they are collected into one view than might have been supposed; and, secondly, that the most distinguished professional men bear witness with an overflowing authority in favour of a course of education in which to train the mind shall be the first object, and to stock it the second. Man is to be trained chiefly by studying and knowing man; and we are prepared for knowing man in life by learning him first in books, much as we are taught to draw from drawings before we draw from nature. But if man is to be studied in books, he will best be studied in such books as present him to us in the largest, strongest, simplest—in a word, the most typical forms. These forms are principally found among the ancients. Nor can the study of the ancients be dissociated from the study of their languages. There is a profound relation between thought and the investiture which it chooses for itself; and it is as a general rule most true that we cannot know men or nations unless we know their tongue. Diversity of language is, like labour, a temporal penalty inflicted on our race for sin; but, being like labour originally penal, like labour it becomes by the ordinance of God a fertile source of blessing to those who use it aright. It is the instrument of thought, but it is not a blind or dead instrument; it is like the works in metal that Dædalus and Vulcan were fabled to produce, and even as the limping deity was supported in his walk by his nymphs of so-called brass, in like manner language reacts upon and bears up the thoughts from which it springs, and comes to take rank among the most effective powers for the discipline of the mind. But more important than the quest of professional knowledge, more vital than the most effective intellectual training, is the remaining question of the temper and aim with which the youth prosecutes his work. It is my privilege to be the first person who has ever thus addressed you in the capacity of Rector. (Loud applause.) But without doubt your ears have caught the echo of those affectionate and weighty counsels which the most eminent men of the age have not thought it beneath them to address to the students of a sister Scottish University. Let me remind you how one of European fame, who is now your and my academical superior—how the great jurist, orator, philosopher, and legislator, who is our Chancellor—how Lord Brougham besought the youth of Glasgow, as I, in his words, would more feebly, but not less earnestly pray you, 'to believe how incomparably the present season is verily and indeed the most precious of your whole lives,' and how 'every hour you squander here will,' in other days, 'rise up against you, and be paid for by years of bitter but unavailing regrets.' Let me recall to you how another Lord Rector of Glasgow, whose name is cherished in every cottage of his country, and whose strong sagacity, vast range of experience, and energy of will were not one whit more eminent than the tenderness of his conscience and his ever-wakeful and wearing sense of public duty—let me recall to you how Sir Robert Peel, choosing from his quiver with a congenial forethought that shaft which was most likely to strike home, averred before the same academic audience, what may as safely be declared to you, that 'there is a presumption, amounting almost to certainty, that if any one of you will determine to be eminent, in whatever profession you may choose, and will act with unvarying steadiness in pursuance of that determination, you will, if health and strength be given to you, infallibly succeed.' The mountain-tops of Scotland behold on every side of them the witness, and many a one of what were once her morasses and her moorlands, now blossoming as the rose, carries on its face the proof that it is in man and not in his circumstances that the secret of his destiny resides. For most of you that destiny will take its final bent towards evil or towards good, not from the information you imbibe, but from the habits of mind, thought, and life that you shall acquire during your academical career. Could you with the bodily eye see the moments of it as they fly, you would see them all pass by you, as the bee that has rifled the heather bears its honey through the air, charged with the promise, or it may be with the menace, of the future. In many things it is wise to believe before experience until you may know, and in order that you may know; and believe me when I tell you that the thrift of time will repay you in after life with an usury of profit beyond your most sanguine dreams, and that the waste of it will make you dwindle, alike in intellectual and in moral stature, beyond your darkest reckonings. I am Scotchman enough to know, that among you there are always many who are already, even in their tender years, fighting with a mature and manful courage the battle of life. When they feel themselves lonely amidst the crowd—when they are for a moment disheartened by that difficulty which is the rude and rocking-cradle of every kind of excellence—when they are conscious of the pinch of poverty and self-denial, let them be conscious, too, that a sleepless eye is watching them from above, that their honest efforts are assisted, their humble prayers are heard, and all things are working together for their good. Is not this the life of faith which walks by your side from your rising in the morning to your lying down at night—which lights up for you the cheerless world, and transfigures all that you encounter, whatever be its outward form, with hues brought down from heaven? These considerations are applicable to all of you. You are all in training here for educated life, for the higher forms of mental experience, for circles limited, perhaps, but yet circles of social influence and leadership. Some of you may be chosen to greater distinctions and heavier trials, and may enter into that class of which each member while he lives is envied or admired—

> 'And when he dies, he bears a lofty name,
> A light, a landmark on the cliffs of fame.'

And, gentlemen, the hope of an enduring fame is, without doubt, a powerful incentive to virtuous action, and you may suffer it to float before you as a vision of refreshment, second always, and second with long interval, to your conscience and the will of God. For an enduring fame is one stamped by the judgment of the future, that future which dispels illusions and smashes idols into dust. Little of what is criminal, little of what is idle, can endure even the first touch of the ordeal; it seems as though this purging power following at the heels of man and trying his work were a witness and a harbinger of the great and final account. So, then, the thirst of an enduring fame is near akin to the love of true excellence. But the fame of the moment is a dangerous possession and a bastard motive; and he who does his acts in order that the echo of them may come back as a soft music in his ears plays false to his noble destiny as a Christian man, places himself in continual danger of dallying with wrong, and taints even his virtuous actions at their source. Not the sublime words alone of the Son of God and His apostles, but heathenism too, even while its vision was limited to this passing scene, testifies with an hundred tongues that the passing scene itself presents to us virtue as an object and a moral law, graven deeply in our whole nature, as a guide. But now, when the screens that so bounded human vision have been removed, it were sad, indeed, and not more sad than shameful, if that being should be content to live for the opinion of the moment who has immortality for his inheritance. He that never dies, can he not afford to wait patiently awhile? And can he not let faith, which interprets the present, also guarantee the future? Nor are there any two habits of mind more distinct than that which chooses success for its aim and covets after popularity, and that, on the other hand, which values and defers to the judgments of our fellow-men as helps in the attainment of truth. But I would not confound with the sordid worship of popularity in after life the graceful and instinctive love of praise in the uncritical period of youth. On the contrary, I say, avail yourselves of that stimulus to good deeds, and, when it proceeds from worthy sources and lights upon worthy conduct, yield yourselves to the warm satisfaction it inspires; but yet, even while young, and even amid the glow of that delight, keep a vigilant eye upon yourselves; refer the honour to Him from whom all honour comes, and ever be inwardly ashamed for not being worthier of His gifts. But, gentlemen, if you let yourselves enjoy the praise of your teachers, let me beseech you to repay their care, and to help their arduous work by entering into it with them, and by showing that you meet their exertions neither with a churlish mistrust nor with a passive indifference; but with free and ready gratitude. Rely upon it they require your sympathy, and they require it more in proportion as they are worthy of their work. The faithful and able teacher, says an old adage, is *in loco parentis.* His charge certainly resembles the mother's care in this, that, if he be devoted to his task, you can measure neither the cost to him of the efforts which he makes, nor the debt of gratitude you owe him. The great poet of Italy—the profound and lofty Dante—had had for an instructor one whom, for a miserable vice, his poem places in the regions of the damned; and yet this lord of song—this prophet of all the knowledge of his time—this master of every gift that can adorn the human mind—when in those dreary regions he sees the known image of his tutor, avows, in language of a magnificence all his own, that he cannot, even now, withhold his sympathy and sorrow from his unhappy teacher, for he recollects how, in the upper world, with a father's tender care, that teacher had pointed to him the way, by which man becomes immortal. Gentlemen, I have detained you long. Perhaps I have not had time to be brief; certainly I could have wished for much larger opportunities of maturing and verifying what I have addressed to you upon subjects which have always possessed a hold on my heart, and have long had public and palpable claims on my attention. Such as I have I give; and now, finally, in bidding you farewell, let me invoke every blessing upon your venerable University in its new career; upon the youth, by whom its halls are gladdened, and upon the distinguished head and able teachers by whom its places of authority are adorned.

Turning now to other events of the quarter which come within the scope of our Retrospect, we would note, first, a remarkable article of the *Times* (May 3) upon the persistency of the Irish emigration. The psychological aspects of the subject, as indicated by the writer, are of singular and grave interest, and while reading we seem as if we were looking forwards into the probable future of a race:—

"The Irish emigration still continues, at a rate which threatens results far beyond the calculations of the economist, perhaps even the wishes of the statesman. It is no longer the overflow of a vessel full to repletion, but the operation of a syphon which drains to the very bottom. If that syphon may be regarded in any visible form it is the railway system, which in the eyes of every Irishman appears to have one common terminus across the Atlantic. He sees trains of hopeful, if not happy, faces going off to the Land of Promise, from which relations and friends have sent not only invitations but the means of accepting them. A train starts to catch an emigrant-vessel as regularly as in England to catch a steamer across the Channel. The emigrant-ships have no longer to peep into every little port to pick up their passengers. They assemble at Cork, and pass in a continuous stream, if it may be so called, across that ocean, which, wide as it be, is easier to an Irishman than the gulf which divides him from England. At present it cannot be said that there leaves Ireland as much as the natural increase, but the causes in operation are not unlikely to make it exceed that rate. As the small holdings are thrown into larger, and the farms grow to the English scale, there must be numbers everywhere bred to the occupation of land, and with all the ideas adapted to it, but unable to get holdings that will require little or no capital. They go across the Atlantic as a matter of course. Brothers, uncles, and neighbours have gone long before, and send, not only good news, but the substantial pledge of its truth in the shape of orders on Irish banks. In Ireland the remark is that these are welcome to go. They are the Irish surplus. They constitute the store-house of independent enterprise which Providence would seem to have prepared through long ages for the peopling of the New World. But there is a class who are not bid "God speed" quite so cheerfully. Labourers—that is, men with strong sinews and thews, who can do a good day's work, and are content to receive wages, are, as they always have been, the chief want of Ireland. The new race of farmers do not like to see them go. But who can pick and choose in human affairs? There are good, easy souls, who enter life with this speculation, who expect in everything the fruit without the husk, the meat without the bone, the sweet without the sour, the harvest without the tillage. In Ireland they expect a good farm, a good house, a good landlord, and some good labourers, who shall come when wanted and do a good day's work. But the postman knocks at all doors, and brings to these, as well as their prouder neighbours, letters and remittances, and good accounts from the Western States; so off they go, leaving the new tenant farmers to manage as well as they can.

"If this goes on long, as it is likely to go on, Ireland will become very English and the United States very Irish. When an English agriculturist takes a farm in Galway or Kerry he will take English labourers with him. This we shall come to at last, strange as it may now seem. The days may, indeed, come when Ireland will be no more Celtic than the Scotch Lowlands are Saxon, the Eastern Counties Danish, Cornwall even Phœnician, and Ireland itself Milesian or Spanish. But several millions more undiluted Celts cannot be poured into the United States without leavening them even more strongly with that very marked element. There will be more poetry, more eloquence, more fanaticism, more faction, more conspiracy, more resentment, more bloodshed, more insubordination, more of the narrow politics that take their origin from race and stop short of society, that ever account the whole less than the part, and think the best use of government is to do convenient ill. So an Ireland there will still be, but on a colossal scale, and in a new world. We shall only have pushed the Celt Westwards. Then, no longer cooped up between the Liffey and the Shannon, he will spread from New York to San Francisco, and keep up the ancient feud at an unforeseen vantage. We must gird our loins to encounter the Nemesis of seven centuries' misgovernment. To the end of time a hundred million people spread over the largest habitable area in the world, and confronting us everywhere by sea and by land, will remember that their forefathers paid tithe to the Protestant clergy, rent to absentee landlords, and a forced obedience to the laws which these had made. Possibly a darker and more turbulent era at home may intervene to efface these Old World recollections. But, even though the vengeful Celt should forgive and forget, that will not prevent the surer development of an intractable race and untoward circumstances in the character of the great American nation. It will be more than half Celtic. Saxon, Dane,

Gael, French, German, African, and other races will be there, but the preponderating element will be that which has risen to its perfection and glory on the banks of the Seine, and fallen to its depth and despair on the western promontories of Ireland. As 'the child is father of the man,' so have we seen nursed and educated by our side at home the power that will dominate over the New World, show its influence over either ocean, and be the lord of a whole hemisphere. This is the true and final home of the Celtic race. It is for this that it has wandered and suffered these two thousand years; for this, that it has never planted the firm foot of civilization on the soil that was not to be its resting-place, but has dwelt in tents and hovels, and not possessed the soil under the soles of its feet. We have been owners and masters of Ireland that its inhabitants might one day have elsewhere a grander possession and rule.

"But what will be the reaction upon us that remain behind? The present natural rate of increase in these isles would take three times the present rate of emigration to bring it to a sandstill. We have to suppose, what, indeed, is not unlikely, that with the growth of the United States and the British colonies, and with the increased and more rapid means of communication, more and more of our people will leave these shores. But wealth and opportunities will still increase at home. Machinery will supply the working power which ever requires the hand of man to guide it; and, while multitudes leave, iron feet and iron fingers will multiply at home. These are servants that rebel not and love not; fit for cold masters; that will not fly, and need neither love nor justice. So we shall have, perhaps, in these islands that peace which we have long desired, if indeed our neighbours will but leave us alone. Most probably, too, as the Englishman supersedes the Irishman in the open market of labour, in our fields and our streets, there will be more order and more subordination to the rights of the proprietor and the employer. But it cannot be expected that we should escape the inconveniences of a household from which the stronger sex, the stronger age, the stronger hands, and the stronger will are ever flying. The community left behind will suffer, probably, more than now, the disparity of the sexes, the burden of the weak and improvident, and the incubus of those who sit on society and demand to be supported. We are an old and burdened State, but we shall be older and shall have heavier burdens still before we have done. There are other encumbrances besides armies and navies, and civil services, and church establishments, that accumulate upon old States, devour their substance, and hamper their movements. There is the universal depredator; the consumer, and nothing else; the race for whom institutions were founded of old by pious fools, and for whom benevolence is this day besotted. Who is there that stands but within speaking distance of the avenues of preferment who does not know the monster evil of an old and wealthy State? There are, indeed, such 'unclean birds,' we dispute not with Mr. Bright, that settle on the branches of the social tree, and devour or spoil the fruits of industry and virtue. But we need not go within the walls of Parliament to learn what and where they are. They are everywhere; they infest all classes; they devour, they invade, they molest; they paralyze, they exhaust. This is the parasite at the table of the rich man which constitutes the chief bane of a high civilization and a fixed state of society. We are throwing off Agitators and Repealers, Socialists, and perhaps Reformers; old England turns itself on its bed and expects another slumber. But its own morbid growth of idleness, luxury, pride, and vice it cannot so easily get rid of. They must grow upon it, all the more from the absence of the more violent annoyances that but lately formed the staple of its domestic annals.

Of different, but not less, and perhaps more immediate interest than the possible results, psychological and general, of the Irish Exodus, are those events of the quarter which may serve to throw light upon the groundwork of popular credulity and superstition. Standing head and shoulders above all events of this kind, during the tri-mestral period, is the history of the doings at the notorious Agapemone, in Somersetshire. Certain proceedings in one of our courts of law have brought the inner-workings of the so-called "Abode of Love" once more before the public. We had intended to have reprinted in this number of our Journal the evidence tendered in court, this being of no small importance to the psychological physician. The judgment, however, not having been delivered we are compelled to postpone our report of the trial. In the meantime, the following summary of the history of the Agapemone, will be read with interest :—

"It is now some twelve or thirteen years ago that a quiet parish in Somersetshire was astonished by the arrival of a clergyman who professed strange doctrines, and was accompanied by strange disciples. According to the chief of this new sect a fresh religious epoch had opened on the world. We were to live under a new dispensation, which, if it did not contradict, was at least to supersede, the forms of belief in which we had all been trained. The keystone of the new system was this :—Various covenants have been at different times offered to man by his Creator. At first Adam was the Divine witness; then the Patriarchs, as Noah and Abraham; then a far greater One than these. But each 'dispensation' was closed whenever any one was found perfect under it. Now, in Brother Prince was found perfection under the Christian dispensation, and, consequently, a new religious epoch commenced, with this man as its witness. Is Prince a religious enthusiast, a lunatic, or an adventurer? It seems probable that he has passed through the first two phases of mental aberration, but we should be not a little curious to know at the present time if Brother Prince believes in himself. The course of his dealing with the property of the dupes whose reason he has perverted certainly affords tolerably strong evidence that he may lay claim to the third character named. If Brother Prince is mad, he has lucid intervals when he can thoroughly well distinguish between a hawk and a handsaw. If at one moment he is the raving leader of a set of religious lunatics, we find him immediately afterwards discussing the intricacies of the share-market with all the acumen of an accomplished stockbroker, and tying up the discretion of his victims in a way which would have won for him the approval of a Lincoln's-inn conveyancer. Brother Prince under one aspect may be a blasphemous buffoon, but under another he is certainly a capital man of business. Being, then, such as we have described, he arrived about the year 1847 at the Castle Inn, at Taunton, with his portmanteaus and his prophetical pretensions. He had been educated originally at Lampeter College, in Wales, and had been ordained Deacon and Priest about twenty years ago. The method of his ministrations, however, does not seem to have earned for him the favour of his spiritual chiefs, for he was successively deprived of his licence, first in Somersetshire by the Bishop of the Diocese, and subsequently by the Bishop of Ely. Being thus an outcast from the regular ministry, in the year 1843 he repaired to Brighton, and there opened a chapel of his own, which he called Adullam, and probably the name was well chosen. In the four following years the ruin of his wits was complete, or his schemes for securing to himself a luxurious and idle existence at the expense of his dupes were sufficiently matured. The scene of his earliest pastoral labours was chosen as the apt spot for the development of his more splendid fortunes; so to Somersetshire he repaired once more with his first followers, among whom four half-witted sisters—the Misses Nottidge—occupied a conspicuous place. With the money he procured from them and others, or, as he would say, with their free-will offerings, he purchased a little property of about two hundred acres. On this he either found a house or built one—we know not how this matter stands—which has since obtained sufficient notoriety under the name of the Agapemone. It was calculated to accommodate some fifty or sixty inmates. There were around it extensive pleasure-grounds, and gardens, and conservatories, and hothouses, and all the appliances of a comfortable country-house. The fee-simple was in Brother Prince—he was not so absorbed in spiritual considerations but that he guarded his private interests carefully upon so capital a point. There was, however, more than this. By some strange mental twist the Prophet had a great fancy for horses and fine equipages. In the Agapemone were to be found horses of great value, both for riding and driving. Brother Prince himself seems to have taken huge delight in driving about the country in a carriage drawn by four horses. The privilege of using this vehicle was occasionally conceded to the disciples, and seems to have been held forth conspicuously as one of the great temporal advantages to be enjoyed by the faithful who had cast in their lot with the High Priest of the New Dispensation.

"Meanwhile strange stories got abroad. Many ladies were received into the Agapemone, and the neighbours believed that the practices of Mormonism might in many particulars be advantageously compared with those of the Agapemonists. There was a public trial some few years ago in which it appeared in evidence, rightly or wrongly, that the Prophet selected female disciples in a manner in which it would be difficult to say whether the ludicrous or the horrible more prevailed. It is hard indeed to believe that Brother Prince was merely a religious fanatic. He instructed his wretched dupes that the judgment had arrived, and that the day of prayer and supplication was over; self-humiliation and self-denial had lost their virtue, and nothing remained but the necessity for pure enjoyment. Men made perfect were to play at hockey. Now, among the earliest of Prince's followers were the four ladies already named. He had known the Misses Nottidge at the second curacy he held, at Stoke, in Suffolk; and when he was driven to his Adullam from that place by the persecution of the Bishop of Ely these ladies went with him. Their names were respectively Louisa Jane, Harriet, Clara, and Agnes. Their father was dead, and he had left to each of them a sum of 5000*l*. or 6000*l*. In 1845, when Prince returned to Charlwich, in Somersetshire, he went by way of Taunton, the Misses Nottidge being of his party, and defraying all the expenses of the journey. When at Taunton, Prince sent for Miss Harriet Nottidge, and informed her that she would be 'giving great glory' &c., by marrying his friend Mr. Lewis Price. Her consent was obtained. Miss Agnes Nottidge was next summoned, and informed that the Spirit had in store for her a great blessing—she was to be married in a few days to Brother Thomas. The wretched lady talked about a settlement in favour of any children she might have by this marriage. Her objections were overruled, and the letter written to her by Brother Thomas on the subject, who signed himself 'her's affectionately in the everlasting covenant,' may stand as one of the most remarkable documents for its unblushing impudence ever known even in the annals of religious imposture. Two days afterwards Prince extorted from the third sister, Miss Clara Nottidge, a promise to marry his follower, one William Cobbe. Thomas and Price were in indigent circumstances at the time, and Cobbe was entitled to a sum of money of his own of about 1000*l*. No settlement was made of the property of any of the three sisters. It was revealed to Prince that the marriages were to take place on the same day, at Swansea, and, what will, no doubt, prove truly appalling to any lady who may read this story, the three brides were to be dressed in black. In July, 1845, the marriages were solemnized at Swansea. Poor Mrs. Thomas seems to have had even at that period some suspicion of the Prophet's true character. She endeavoured to dissuade her husband from obeying a summons which he received from him at Ilfracombe, and which ran thus:—'Brother Thomas, I command you to arise and come to Weymouth. Amen!' The struggle against Prince's influence was continued for a short time, but, as might have been anticipated, was wholly unavailing, and the end of it was that Mrs. Thomas was not allowed to reside with her husband at the Agapemone. In 1846 a child was born to her, and after a sharp struggle Mrs. Thomas and her mother were in 1850 appointed by the then Vice-Chancellor Knight Bruce guardians of the child. Meanwhile Prince had drawn the eldest sister, Louisa Jane, into his toils. In 1846, howevever, this unfortunate lady, who had evinced symptoms of insanity, was placed in confinement, from which she was released in 1848 by the Lunacy Commissioners. On the very day of her release Brother Thomas was waiting for her, and took her down to the City to Prince's broker, to execute a transfer to the Prophet of the stock then standing in her name. There was a momentary difficulty about the precise amount, but very shortly afterwards the transfer was executed. In 1858 Miss Louisa Jane Nottidge died intestate. The question which has been for several days discussed before Vice-Chancellor Sir J. Stuart was as to the validity of this transfer.

The case for the defendant, as very ably stated by the counsel engaged, amounted to this,—that you could not accept any divergence from the religious opinions which are accepted by the majority as positive evidence of insanity. In 1848 Miss Louisa Jane Nottidge, after due investigation, had been released from confinement by order of the Lunacy Commissioners, a competent tribunal for inquiring into and adjudicating upon the matter. She subsequently brought an action against her relatives for false imprisonment in the asylum, and recovered damages against them; therefore, if any weight is to be attached to the previous decisions of judges of sufficient jurisdiction, Miss Louisa Jane Nottidge was not of insane mind at the time of the execution of the transfer. Furthermore, she had every reason—it must be understood that we are but recapitulating the arguments used for the defence—to disinherit those of her own relatives who had been instrumental in locking her up, and in constituting Brother Prince the object of her bounty, inasmuch as he was not only the object of her religious reverence, but he was to provide, and actually did provide her, with all the comforts and luxuries of life. She was then not of insane mind,—she was deeply exasperated against her own family, as deeply interested in favour of Prince,—she was anxious to spend the remainder of her days in the Agapemone, in the society of her sisters Harriet and Clara, Mrs. Price and Mrs. Cobbe, and she was to receive as a *quid pro quo* sustenance and support in this establishment during the remainder of her days. We have given the arguments with full force, because it is earnestly to be hoped, in the interest of public morality, that the Court of Chancery may find it consistent with a due and impartial administration of the law to annul this transfer. It would be a most unhappy conclusion if we were to be told upon the authority of a Vice-Chancellor that as English law stands a religious imposter — a conscientious fanatic, if you will—might legitimately exercise his spiritual influence over his female devotees so as to induce them to denude themselves and their natural heirs and kinsfolk of their property in his behalf. We will drop the question of religious imposture altogether, but what if Dr. Paul Cullen—what if our own Archbishop of Canterbury, had extracted a gift or transfer by religious pressure from a half-witted woman,—ought the validity of such an act to be maintained? Surely not. The weakest point of Prince's case was the one in which he endeavoured to make out that he had personally nothing to do with the transfer—it was Miss Nottidge's free-will offering. Now, when the wretched lady was let out of the asylum whom did she find on the threshold? The chosen disciple of Prince. What did he do with her? He took her straightway down to Prince's broker in the City of London, to execute the transfer of the stock standing in her name to Prince. When the transaction was completed whither did he conduct her, and to whom? To the Agapemone and to Prince —and she lived with him, and under his influence, from the date of her liberation from the asylum until the date of her decease. Yet it was gravely argued thaf Prince could not be held to be cognizant of the transaction. The last thirteen or fourteen years of Miss Nottidge's life, in short, were spent between the Agapemone and the asylum—many will question if she was ever out of a lunatic asylum at all. We do not, of course, affect to discuss this question with the technical knowledge of those gentlemen who have spent their lives in the study and the administration of equity; but as a matter of common sense it will be most deplorable if the silliest and weakest minds of the community are left at the mercy of such men as Prince, and if the Court of Chancery holds that they may apply the religious screw to such women as the Nottidges, and pocket the plunder.—(*Times*, June 12th.)

The introduction, in May, of a Bill into Parliament for the removal of certain restrictions upon the sale of wine in refreshment houses, gave rise to much discussion as to the influence which greater facility of access to wine might have upon intemperance in the country. The moral question is one of no small moment; but in its political aspect, by some strange freaks of partizanship, the question became so waiped, that little respect was shown to those who contended against the Bill from a conviction that it would have the effect of promoting still greater intemperance in the land. On the opposite side it was argued that the substitution of a more innocent liquor like wine for a more highly stimulating and heady fluid like spirit or beer, could not but be advantageous to the community. A similar argument in respect to beer as compared with spirits, if we mistake not, was urged with great effect when the Bill authorizing the establishment of beer-houses throughout the land was debated in Parliament several years ago. Now, in 1854, a Select Committee of the House of Commons reported that—" The beer-shop system was a failure. It was established under the belief that it would give the public their beer cheap and pure; would dissociate beer-drinking from drunkenness, and lead to the establishment, throughout the country, of a class of houses of refreshment altogether free from the disorders supposed to attend exclusively on the sale of spirits." This does not look very promising for the argument on the moral advantages which some suppose may arise from facilitating access to wines. But let us listen to Mr. Gladstone's remarks on the question. (Debate, May 7th.) He said:—

"It is a question whether this Bill will tend to promote and increase intemperance. Who is it that tells us such will be the case? Who are they who combine to form the opposition to this Bill upon the ground that it is likely to increase drunkenness? It is the same proposition, but it proceeds from parties who are singularly united in a sort of concordant discord. There is an old fable, called, I believe, the 'Vision of Hercules,' which is in point.

When Hercules was young he dreamt that he came to a certain point of the road, where he was met by two figures — one the figure of Virtue, and the other the figure of Vice. He was solicited by Virtue to go one way and by Vice to go another. We are in the position of Hercules, as we are encountered by two figures of Virtue and Vice. But, instead of Virtue soliciting us to go one way and Vice pressing us to go another, we have both Virtue and Vice leagued against us, both standing across the road and refusing to allow us to proceed. I know the virtuous motives of those who support the temperance movement, which the hon. member for Marylebone (Mr. James) has manfully thrown overboard in his speech. The arguments used are a group of assumptions fastened together, which it is difficult to separate and to deal with. We are told that the use of wine is to be considered exactly as that of ardent spirits. These practical philosophers will not condescend to draw any distinction; they have invented phrases, 'alcoholic liquids,' 'intoxicating liquids,' and such like; but my right hon. friend the member for Oxfordshire could readily show them the fallacy of mixing up things which are so distinct. There is a difference between the lighter wines of Northern Europe and the gin which is consumed in rivers in our great towns. Some one has given us a deplorable description of the drunkenness that prevails in France, and I begin to think that no English traveller could have made a proper use of his eyes. However, I have found a testimony which is entitled to great weight, coming from a man pledged by his sacred profession, eminent for his eloquence, distinguished and beloved for all his virtues—Dr. Guthrie. That gentleman before he devoted himself to his present calling resided for some time on the Continent, and in one of his sermons he says that he was in Brussels and Paris during periods of great national festivity, and that he did not see in seven weeks as much drunkenness in those capitals as he would meet with in seven short hours in London, Edinburgh, or any other of our large towns. That, Sir, is the testimony of an impartial witness. I have spoken of the fable of Virtue and Vice; and I appeal from them to what I call the common sense of the House of Commons and of the country at large. I have heard references made to the number of petitions presented against this Bill; but I deny that this Bill is disapproved by the public opinion of England. We all know that wherever there is an organization the numbers which it commands are easily available for the purpose of signing petitions; but in this case we have the strongest evidence, from the press, from various authorities, and even from several distinguished prelates, in favour of the principle of the measure. The real question is this—Will you attempt to modify or improve the present system? I grant that this Bill is so far inconsistent with the report of the committee that it falls short of that report, but I hold that it is in harmony both with the spirit and even with the letter of that report. It is insisted by some that you should treat the use of wine, and even of the lightest wine, as you do the use of brandy, for instance. It is also insisted that you have nothing to look at except the number of houses for the sale of liquors in order to ascertain the measure of drunkenness that prevails. The case of Liverpool has been referred to, and I will show the House how untrue that is in the case of Liverpool. It is true there is a large number of publichouses in Liverpool, but I think the hon. member for Leominster (Mr. Hardy) when he was dealing with this subject, and when he was referring to the case of Liverpool and Manchester, did great injustice even to those beer-houses with respect to which so much has been said. He adverted to the great difference in point of sobriety in favour of Manchester against Liverpool, but he omitted to notice that the characteristic of Manchester was that there was a greater number of public houses with a smaller number of beer-houses, and that the characteristic of Liverpool was the reverse. I have got before me the number of persons brought before the magistrates in Liverpool for drunkenness in a series of years, and also the number of licenses granted. I find that in 1846 the magistrates gave seventeen new licenses to public houses; and in 1847 there was a diminution of 256 in the number of persons brought up for drunkenness. In 1852 the magistrates gave no new licenses; and in 1853 there was an increase of 1,144 in the number of persons brought up for drunkenness. In 1854 the magistrates gave two new licenses; and in 1855 there was an increase of 1,018 in the persons taken up for drunkenness. Lastly, in 1857 the magistrates gave the immense number of thirty-two new licenses in Liverpool; and in 1858 there was a decrease of 1,259 in the persons brought up for drunkenness. That shows you how loosely and how widely these doctrines are thrown out. Some hon. members may have seen a small tract in which a great number of eminent medical men in this country are made to declare that all strong liquors are extremely mischievous, and that total and universal abstinence from alcoholic drinks and intoxicating beverages of all kinds would greatly contribute to the health and prosperity of the human race. Naturally enough, I looked among the list of names for that of the gentleman of whose professional assistance I had availed myself, and by whom I have been recently advised. I found his signature appended to the document, at which I was not a little surprised, seeing I remembered that he recommended me, as a means of recovering my strength, not illiberal potations. I afterwards asked him whether he had signed that document or not; he replied that he had, and on expressing my surprise at his having done so, he assured me that in signing it he meant nothing more than that excess of water was less injurious than excess of wine. That was the opinion of a very eminent medical man, Dr. Ferguson; and I believe it is a gross error to suppose that the testimony of those who study the health of mankind is against the moderate use of spirituous liquors. It is said by some that there can be no such thing as a moderate use of them. But is not the love of money, for example, as prevalent and as universal as the love of wine? I never heard that those who denounced the use of alcoholic liquors carried their own principles with consistency into effect; and if they did so, the end of it ought to be that they should go, like the Anchorites of old and people the deserts of Egypt. But the doctrine, that the use of wine is to be treated as an unqualified mischief, and almost as a sin, is incompatible with the usages and necessities of society. That, however, is at the root of the opposition made by one portion of the opponents to this Bill."

This is not very satisfactory; but the question is now submitted to experience, and we apprehend that the majority of individuals will not trouble themselves with any other view of the subject, than that it is a very pleasant change to be able to get light wine at a reasonable rate, without the, until now, almost necessary associations of beer and spirits; an advantage that we are by no means disposed to murmur at.

From the morality of wine-drinking to the morality of the lower classes of our great towns is an easy step. The following illustration of certain phases of life among the metropolitan lower classes is instructive:—

Mansion-house.—John Keating, an Irish shoemaker, one half of whose features was obscured by hair, and the other half by dirt, was charged with assaulting Ellen Lawler by stabbing her in the face with a knife.

The prosecutrix, the left side of whose face exhibited a huge circular patch of sticking plaster said, "Plase yer Honours, my name's Ellen Lawler."

Mr. Goodman (chief clerk).—And what is your husband's name?

Prosecutrix.—Pat Bresshanan.

Mr. Goodman.—Then I suppose your name is Bresshanan?

Prosecutrix.—Lord, me, yer Honour, my name is Ellen Lawler.

Mr. Goodman.—How can that be if he's your husband? Your maiden name, you mean, I suppose, was Lawler?

Prosecutrix.—Yes, my maiden name, and my name now; for, though I call Pat my husband, we ain't married, but we've lived together a long while, and he's a good husband, too.

Mr. Goodman.—Why did you not say at first that you were not married? Go on with your evidence.

Prosecutrix.—Well, on Saturday night I was sitting at supper with Pat, when who should come in but Moggy Qunk; so, says I, "Sit down, Mog, and have some supper." But, yer Honours, she was nasty, and got to words, and at last says she, "If you are a woman, come down into the yard and have it out." "Very well, Mog," says I, "I'll come down, cos you know I ain't afraid of you;" and she says, "No, I knows that, I knows you are too good a woman for me;" and that's right, yer Honours, because, though I'm a quiet woman, I can stick up for myself like a good 'un, only I didn't want to hurt her nor nobody. Well, when we got downstairs we quieted, and we wasn't a-going to fight; but there was a mob at the door, and that there prisoner there made a rush at me, and gave me a job in the mouth with his fist, which loosened all my teeth, and then he jobbed at me again with something in his hand, which I took to be a knife, and stabbed me in the face. So I calls out, "Pat," says I, "he's made a hole in my face with a knife, and I'm kilt intirely!" and then down I went, while Pat dashed out and collared him. The hole ain't quite through my cheek, but it's a whacker, and I hopes your worship will give me purtection.

Pat Bresshanan said—When that man stabbed my wife—

Mr. Goodman.—She says she is not your wife, though she lives with you.

Witness.—So she does; and an honest woman she is.

Sir R. Carden.—Why don't you make an honest woman of her?

Witness.—I don't mean in that way; but I mean that she's too honest to meddle with anybody's business.

Mr. Goodman.—She can fight well, at all events, according to her own confession.

Witness.—She's a regular good'un, yer Honour; she's a rale beauty, and no mistake.

Sir R. Carden.—Then why don't you marry her?

Witness.—Well, I will, yer Honour, I will. But about this here prisoner. When I rushed out upon him he struck me, and I'm sure I felt a knife in his hand.

Prisoner.—My Lord, it wasn't a knife at all, and but for the drink that was in my head I shouldn't have done it. That woman had been throwing some water over me just before, and when I went out again I took a little poker with me, and I hit her with that. It was a very little one, yer Honour, but sharp at the end.

Sir R. Carden.—A poker would not have cut like a knife. However, knife or poker, I shall send you to prison for twenty-one days.

Prosecutrix.—And, oh! yer Honour, wont you bind him over to keep the peace towards me?

Sir R. Carden.—The police will protect you. I'd rather bind you over not to live with that man again till you are married.

Prosecutrix.—And, faith, yer worship, I'd not object to that.

Pat Bresshanan.—And, yer Honour, I will marry her. I've often talked about it, and now I'll do it.

They then left the Court.—*Times*, April 10th.

It is by no means an easy task to exercise a moral influence upon the classes of the metropolitan population, of whom Pat Bresshanan and his concubine are examples. We are not, however, doing what we might in this matter. Far from it, indeed; and we may derive some very useful hints from sundry comments of the *Times* (April 16th), on a Pastoral Letter addressed by the Bishop of London to the laity of the metropolitan diocese, at the beginning of the quarter:—

"The Bishop appeals very earnestly to the claims which the poor have on those who live by their labour; claims substantially the same as those which have fringed gentlemen's parks with picturesquely-constructed cottages, and made the village church an almost necessary complement of the landscape. Employers have only to hunt out their men in alley and court, and they will find families as interesting, and as much in need of religion, as that at the pretty lodge or the model farm. All this is strictly and sternly true. The relations of property and labour are the same everywhere, and were logic the rule of life there would be no need for Pastoral Letters. But the simple fact that things are not as they ought to be, and that the Diocese of London is not in the same state as the rural districts of the favourite counties, sends us back to inquire how this comes to pass. We must not expect that all people will act by Rule of Three. If they act differently it is because the circumstances are different, and it becomes us then to adapt our plans to those circumstances. Vain it is, we grieve to say, to raise up moral visions in a crowded and dingy metropolis. The squire and his lively sons, the lady and her kind daughters, the pastor and the parsonage, and all the rest that, barring a few ruffles and an occasional hitch, goes on so smoothly on the estate of a great proprietor, —all have a secret and a charm of their own. The chief secret of the whole consists in a warm mutual interest, and distinctly defined social relations. Power, authority, influence, dependence, order,—in a word, nature itself, blend all into one family, in which the landlord or the clergyman feels a real and natural interest in the people, only second to that he has for his own wife and children.

"Far different is the relation between employer and employed, or landlord and tenant, when the one party knows not even the faces, names, or localities of the other, and only regards them as dishonest and degraded beings, practising every art of conspiracy and imposture to evade lawful claims on their labour or their purse. Far different when the employed are only a class, changing their masters, their habitations, and, too often, their companions, from year to year, if not oftener. Unfortunately, the hackneyed phrase of the philanthropist who appeals to us for the 'masses' is too true to the fact. There are masses that we have to deal with; rude masses; uninviting masses; human nature in oceans and swamps, rather than rivers and lakes. It is a great Dismal Swamp of human existence for which the Bishop pleads. This is the true scene of modern martyrdom. A colony at the Antipodes, a Northern County, the Punjaub, Jamaica, Central Africa, and British Columbia, all have their charms for this or that temperament or period of life. There is scarcely any position that may not be sweetened with matrimony and 1000*l.* a-year. Even the Parliamentary curate's stipend of 80*l.* is wealth and happiness to a young bachelor who can ride colts, or has good health. Suffering and death themselves may be made attractive by picturesque circumstances and the prospect of a well-written biography. You will not live to lose the manners of society, or return home to be detected in Fejee provincialisms. Far otherwise is it to the man who devotes himself to the pastoral duties of a clergyman, or even a good neighbour, in a poor and populous metropolitan district. He sacrifices everything,—fortune, health, connexions, manners, tone, aspect, cheerfulness, with the whole exterior,—ay, more than the exterior of the English gentleman. When the Apostle wished himself accursed for the Jews, he could not have imagined an earthly lot in which the sacrifice could be so nearly consummated. The man who spends his days among the London poor, and his nights for them, may acquire the odour of sanctity, but he will lose that of society. Even among the saints he can hardly expect to shine so bright or cut so good a figure as the man who has ministered in handsome churches and splendid drawing-rooms to the wants of a West-end congregation.

"All experience shows that a population of this sort, and in this condition, cannot be dealt with as the simple folk within sight or sound of a village church, or even the small knot of gentry and tradespeople in a country town. In those vast metropolitan parishes,—three exceeding 35,000,' says the bishop; four more exceeding 30,000; five more exceeding 25,000; six more exceeding 20,000; and so on,—altogether sixty-six parishes exceeding 10,000, we have a chaos of social elements, a dead level of conditions, a mere undeveloped mine of moral qualities. This is not the case for individual agencies, marvellous and even miraculous as they have proved in some emergencies. Ordinary men cannot breast such waves, and even extraordinary men may fail. The Bishop hails missionary enterprise, but hopes more from any scheme for subdividing parishes into manageable districts. The latter is the work to be promoted by the Diocesan Church Building Society. Of course, there must be both churches and clergymen, and we hope eventually to see them adjusted one to the other. But, as there are not churches, or the churches that are, so we are told, are but ill-attended; as clergy engaged in this hard service require mutual help and countenance; as the spiritual war is, in fact, the invasion of an enemy's territory, we cannot help thinking the Bishop would get more sympathy, more money, and more men for a well devised missionary work, framed to the scale of the whole metropolis, than for more 'churches,' in the vulgar sense of the word. The handsome church, on a costly site, with its actual or threatened tower, its permanent endowment, and its staff of petty officers, is the Church's three-decker, on which we spend so much, and so often find to be useless. What we really want is the flotilla of gunboats, to push into lanes and alleys. We want something more locomotive than church and steeple; more winning even than reredoses and copes; sweeter than church bells; and more penetrating than either the feet or the eloquence of dignified rectors. If the Church does not adapt her means to the end, and make it a 'day of little things,' Dissenters and even Roman Catholics will. In fact, this is what they are doing; and this it is that enables them to make up for their immense disadvantages in social rank and position."

Several instances of suicide have been recorded by the daily and weekly journals within the three months which have just transpired, of great interest to the practical psychologist, not so much from possessing any unusual feature, but from the light which the history of the cases throws on the motives leading to ordinary every-day—in short, common-place suicide. We shall terminate our Retrospect by laying the cases before our readers simply as they have been reported in the papers, chiefly the *Times*:—

Guildhall.—Ann Ginsty, a decent-looking woman, was charged with attempting to commit suicide by swallowing a quantity of laudanum.

Brown, 156, said he was called to a room on the second-floor, at 3, Charlotte-court, Redcross-street, where he found the prisoner suffering from the effects of poison. He saw an empty bottle labelled laudanum on the table, and she said she had taken it all, alluding to the previous contents of the bottle. He procured medical assistance, and as soon as the prisoner was in a condition to be removed, he took her to the police-station.

Alderman Hale.—Is this the first attempt she has ever made upon her life?

Officer.—No, Sir; she made a similar attempt about six weeks ago.

Alderman Hale.—And what is the cause of these attempts to destroy herself?

Springate, the gaolor.—She tells me, your worship, that it is jealousy of her husband, and she has been on her knees all the morning praying for forgiveness.

The husband here came forward and corroborated the gaoler's statement.

Alderman Hale.—How long have you been married to the prisoner?

The husband being unable to answer the question, the officer supplied the information, stating that they had been married only eight months.

Alderman Hale (addressing the prisoner) said,—You must be a very foolish woman to poison yourself because your husband is unfaithful to you; and let me remind you that the offence you contemplated is a most serious one, for the commandment, "Thou shalt do no murder," applies equally to the taking of your own life as that of another.

Prisoner.—I will never do the same again, Sir. I will pray for forgiveness.

Alderman Hale.—Very well. Upon that promise I will let you go home with your husband; but if you are brought here again on such a charge, you will most assuredly be tried at the Old Bailey for it.

Mansion-house.—Sarah Johnson, a decently-attired young woman, was charged with attempting to destroy herself by taking laudanum in the ladies' waiting-room at the Blackwall Railway station.

Police-constable 519, said,—Yesterday afternoon, about four o'clock, I was on duty at the Blackwall Railway station, when I was called to the ladies' waiting-room and told that the prisoner, who was sitting there, had been taking poison. I asked if such was the case, and she said Yes; she had taken sixpennyworth of laudanum because she was so tired of her life that she could live no longer. I at once sent for a medical man, who gave her an emetic, which had the required effect, and I afterwards conveyed her to the station.

The prisoner, when asked what she had to say, replied, "Nothing."

Police-constable, 519.—Her husband is here, my Lord.

A very respectable-looking young man ascended the witness-box and said,—The prisoner is my wife, my Lord. We have been married for about three years, and for the first part of the time we lived very happily together, till an improper intercourse took place between her and my master, who is a very respectable tradesman, not far from here. It had been carried on some time when I had a suspicion of it, and one night when I went home I asked her about it and she confessed to the whole. I immediately applied to a solicitor, but he told me that, as I had no witnesses to the adultery, I could take no legal proceedings. I then went to my master, who offered me a sum of money to settle it, and, as I found I had no legal remedy, I accepted the money to compromise it, and took my wife home and tried to make her comfortable again. But, of course, after what I had discovered, we had a few words now and then, and on the 30th of January last she left me, after we had quarrelled, at eleven o'clock at night, and I have not seen her since till now.

The Lord Mayor.—Have you any family?

Husband.—No; no family.

The Lord Mayor.—Have you anything to say, prisoner; you have heard what your husband has said—is it true?

Prisoner (crying.)—Yes, quite true.

The Lord Mayor (to the husband).—Do you feel disposed to take her back and try her again?

Husband.—Under the circumstances, I cannot do so. I think I have given her one very fair trial; and besides, I had provided her with plenty of money and everything necessary for comfort, and I supposed myself to be entirely out of debt; but after she left me I found I had a good many bills to pay.

The Lord Mayor.—I have nothing to do with that; but I have no doubt that she is a bad and depraved woman, and it is my duty to send her to prison for seven days.

Prisoner.—Richard! Richard!

Husband.—Will your Lordship remand her that she may go back to her father?

The Lord Mayor.—Has she a father?

Husband.—Yes; a man in a very respectable position.

The Lord Mayor.—Then, at your intercession, I will remand her for a few days; and I hope her father will come forward to take care of her.

The prisoner was then remanded, and her husband, by permission, went below to speak with her.

Worship-street.—Ellen Norton and Mary Anne Hodges, two well-grown girls, were charged with attempting suicide.

The girl Norton is the daughter of a brass-worker in New Norfolk-street, Shoreditch, and having been found by her elder sister to have formed an acquaintance with very disreputable characters, she very properly pointed out the consequences to her, and rebuked her for it. This seemed to have had no effect; and as the girl frequently stopped out late at night, occasioning her family great unhappiness, this reproval appeared to have been as frequent; and the consequence was that, instead of the prisoner mending her ways, she stayed out late again the night before this charge, came home so ill that a doctor was sent for, and it was then discovered that she had swallowed a large dose of muriatic acid, used by her father in his business, and the bottle usually containing it was found to have been emptied. Clarey, a constable, was sent for, who found her, at half-past two in the morning, fearfully exhausted and sick, and altogether so ill that she could not be moved till late the next morning, when she was taken into custody for her own protection, and to see what could be done with her.

The prisoner acknowledged to the magistrate that she had attempted to kill herself merely because she had been blamed for stopping out late, and seemed to consider herself rather ill-used than otherwise; but

Mr. Mansfield told her that such conduct as hers was depriving her of all chance of becoming a respectable or happy woman, and committed her to prison for a week that she might derive advantage from the advice of the chaplain.

In Hodges's case, Archer, of the H division, was fetched to the house of her father, in Winchester-street, Spitalfields, at three o'clock that morning, and there found the prisoner totally senseless, and dying, as her mother-in-law said, from poison she had taken. A surgeon was procured, who succeeded in restoring the prisoner to consciousness; but, instead of repenting of her folly, she repeated to the officer her fixed determination to make away with herself.

The prisoner's father, who was so affected that he was unable to speak for some time, and then did so in tears, said he had unfortunately lost his wife some time ago; and, as he had a family to care for, he married again last September. This seemed to have given the prisoner great offence. She became so jealous of her step-mother that it was almost impossible to do anything with her; and he could only suppose she must have been labouring under insanity, arising from this jealous feeling, when she committed this act, as she had never shown such a disposition before, and was otherwise a well-conducted, industrious girl.

The prisoner said she had had a quarrel with her step-mother, and, suddenly seizing the bottle of poison from off the sideboard, swallowed the contents. She accused her step-mother of harshness to her, and

Mr. Mansfield, though condemning the prisoner's conduct, for which her father's second marriage formed no justification at all, still thought it better, for the happiness of all the family, that the girl should be removed from her home, and maintained by herself elsewhere, and that some arrangement of this kind might be effected, remanded the prisoner for a few days that she might also have time for reflection.

An inquest was held yesterday afternoon (June 1), at the Guildhall, Plymouth, before Mr. John Edmonds, the coroner for the borough, to inquire into the circumstances attending the death of Mary Anne Luke, who died on Wednesday evening from injuries she sustained by jumping out of a window on the previous afternoon.

The case having been much talked about during the day, there was a very large crowd in and around the Guildhall. The hall itself was completely filled, as were also its approaches, and there were hundreds in the street who could not get in.

The Coroner addressed the jury, and said they were called together for the purpose of inquiring into the circumstances attending the death of Mary Anne Luke. This investigation was of considerable importance, not only on account of the station of the deceased, but also as to how far the fatal occurrence was attributable to the conduct of the father. By the rule of the law, a parent might chastise his child properly, but that must not be done violently. It seemed from what he had heard—but they would have the evidence by and by—that this young woman, the deceased, was violently beaten with a rope, and that she fled in consequence from her father, and jumped out of a window. By law, if one person, under a well-grounded apprehension of violence from another, had to resort to such means as that by which this young woman met her death, he who had caused it was held responsible.

The jury then adjourned to the house to view the body.

When Thomas Luke, the father of the unfortunate girl, came into court, he was received with yells from the assembled crowd.

Mary Abbott, the first witness called, was examined, and said,—I am a widow, and reside at Mr. Thomas Luke's house, 30, Union-street. Mr. Luke and all his family reside there. I am employed by him as housekeeper. The deceased was his daughter, and was single; her age was eighteen, and she was a healthy girl, except during the last few months. Her employment was keeping her father's business and selling boots and shoes. Mr. Luke has a wife and five other children, the deceased being the eldest. All the family lived in the house, with the exception of Mr. Luke and the eldest son, who took their meals at one of the other shops, Mr. Luke having four in different parts of the town. All the family slept at the house in Union-street. At two minutes to nine on Tuesday night last, Miss Luke came home to the shop with her mother, having been at the establishment in Bedford-street all the afternoon, where she went with her father. They appeared to be good friends. She went up to her bedroom, which was two stories high, to take off her bonnet and shawl. She came down again on her sister calling her, and went out into the kitchen. Her father was there, and insisted on an explanation, which she said she could not give, and he then slapped her face with his hand. He might have given her two or three slaps, but I cannot say. She cried, but said she could not give any explanation. Her father then took a piece of line and struck her.

[The "line" was handed to the coroner. It was apparently a piece of clothes-line, about the thickness of a small finger. As it was exhibited, a roar of execration burst forth from the densely-crowded court, which the coroner appeased by requesting the crowd to desist from any exhibition of feeling, whatever their thoughts might be.] Her father went into the yard and cut it from a clothes-line. He held it in his right hand when he came in, and the deceased was standing up. No one had hold of her. I think the line was once doubled, and her father struck her with it on the arm. I do not think more than two or three blows were struck. She held up her arm, receiving the stripes on it, and said, "Don't, father." He ordered her to her bed, and she went. That was instantly after he struck her the last blow. She was crying then, but not much, and left the room quickly. No one followed her. The kitchen door was then shut. When Mr. Luke ordered his daughter to bed, he threw the rope down on the floor. He did not threaten to beat her again, but sat down, and she ran up-stairs, and I stood at the kitchen table. We heard the deceased's window thrown open, and her sister Emily screaming that deceased was out of window. I ran into the court with Mr. Luke, and there we found her on the ground, quite insensible. She was lying on her back, with her head thrown on one side. She was removed indoors, and I went for a surgeon, Mr. Whipple. Deceased died at ten minutes after eight o'clock last night. Deceased spoke yesterday, and she was attended by Mr. Whipple and Mr. Square.

Mr. Whipple, surgeon, was the next witness examined. On Tuesday evening he was called to attend the deceased, and found her in the kitchen lying on a sofa. Deceased was lying on her back, and there were two wounds from which blood was flowing—one in the temple and one in the forehead. Blood was also flowing from both nostrils. He enlarged one of the wounds for the purpose of minutely examining the skull, and then found that a portion of the frontal bone had been separated and driven down on the brain. Being satisfied that the case must terminate fatally, he sent for Mr. Square, as in case any grave charge should be brought against any person, it would be satisfactory to have the evidence of two medical men. He told Mr. Luke there was no hope, and he appeared quite distracted, and stated that he had chastised her severely—very severely. Witness attended the case, and deceased died the previous evening. She was never sufficiently conscious to be examined by a magistrate; she could answer "Yes" or "No," but was not capable of reflection. A person jumping out of a window twenty or twenty-five feet high, in case of the head coming to the ground, would be likely to receive such an injury as the deceased did. The cause of death was fracture of the skull and laceration of the brain. Since the death he had examined the body, and then found several contusions produced by the fall; on the left arm near the shoulder there were three or four stripes, similar to what would be produced by blows of a stick or cord. They were not very severe and the skin was not broken.

Emily Luke said she was sister to the deceased, and was about fourteen years of age. She detailed the particulars of the altercation in the kitchen, and said that when her father ordered deceased to her room, he shut the door, and, going back by the fire, sat down. Witness went out into the garden, and before she thought it possible for her sister to have reached her bedroom she heard the window thrown up. She looked upwards, and saw her sister spring out. The next instant she was lying on the ground, having fallen on her head. The witness also testified to the kind manner in which the deceased was invariably treated by her father. In answer to a question as to how some glass became broken, she said her sister leaned against the window while her father was beating her, and the rope struck one pane of glass and broke it. She could not remember the time when her sister had been punished by her father before Tuesday evening. The words used by deceased to her father in the kitchen were—"I have nothing to say; what can I say?" The Coroner, assuming this witness had concluded her evidence, told her she could withdraw, whereupon she said she had something "particular" to communicate. She was requested to proceed, and made the following extraordinary statement:—"About ten days or a fortnight before this happened, my sister showed me a bottle of oxalic acid and a bottle of laudanum, saying, 'I shall take it if father is told.' I understood her meaning to be, that she and the shopman had not been acting as they ought. On Tuesday evening, just after she came home, father sent me up to her room to request her to come down to him. She then said, 'I took a large dose of oxalic acid in the dinner time, and I am surprised that it has taken no effect.' This was before father thrashed her. I saw a bottle of poison taken from her pocket after she jumped from the window."

Mary Ann Avent deposed,—I am a shopwoman with Mr. Luke, at 30, Union Street. On Tuesday morning I complained to Mr. Luke that I was not comfortable in his shop, and gave him notice to leave. I have seen the foreman and deceased eating together in the shop, and thought it improper. On Tuesday morning I told deceased of it in the presence of her father, and she said nothing. The man came into the shop, and Mr. Luke discharged him on the spot, promising him his regular wages if he would call the two following Saturday nights.

The Coroner having summed up, at twenty minutes after ten the jury retired to consider their verdict.

After an absence of half an hour, the jury returned into court with the following verdict:—"That on the 27th day of May, in the year of our Lord 1860, the said Mary Ann Luke, having been severely beaten by her father, Thomas Luke, of Plymouth, at No. 30, Union-street, within the borough, became excited, and, while labouring under temporary derangement, jumped from the bedroom window of her father's premises and fell on the ground below, whereby her skull was fractured and brain lacerated. That she languished therefrom, at 30, Union-street aforesaid, until the 30th day of the said month of May, and then and there died of the said fracture of the skull and laceration of the brain, and the jury further say that her said father's conduct towards the deceased was marked by undue severity."

During the time the foreman was reading the decision of the jury, Luke made use of some incoherent expressions, and then sank forward on the desk weeping like a child.

